# Supplementary material for: P-TRAP: a Panicle Trait Phenotyping tool
Source: BMC Plant Biol. 2013 Aug 29;13:122. doi: 10.1186/1471-2229-13-122 (PMC3848748; doi:10.1186/1471-2229-13-122)
Supplement: Additional file 7 — P-TRAP source code. The Netbeans project that contains the source code of the application. [file 1471-2229-13-122-S7.zip › javadoc.zip!/javadoc/org-basmati-workspace/index-all.html]

Index (WorkSpace)


JavaScript is disabled on your browser.


- Package
- Class
- Tree
- Deprecated
- Index
- Help

*org.basmati.workspace 1.0*

- Prev
- Next

- Frames
- No Frames

- All Classes

A C D E F G H I M N O P R S U V 


## A

actionPerformed(ActionEvent) - Method in class org.basmati.workspace.CollectMasterReportAction


actionPerformed(ActionEvent) - Method in class org.basmati.workspace.ColoredImageAction


actionPerformed(ActionEvent) - Method in class org.basmati.workspace.EditResultFileAction


actionPerformed(ActionEvent) - Method in class org.basmati.workspace.EditViewGrains


actionPerformed(ActionEvent) - Method in class org.basmati.workspace.ExpressRunAction


actionPerformed(ActionEvent) - Method in class org.basmati.workspace.FindGrainsAction


actionPerformed(ActionEvent) - Method in class org.basmati.workspace.FindGrainsSingleImageAction


actionPerformed(ActionEvent) - Method in class org.basmati.workspace.FindGrainTraitsAction


actionPerformed(ActionEvent) - Method in class org.basmati.workspace.FindGrainTraitsSingleImage


actionPerformed(ActionEvent) - Method in class org.basmati.workspace.FindSkeletonAction


actionPerformed(ActionEvent) - Method in class org.basmati.workspace.FindStructureSingleImageAction


actionPerformed(ActionEvent) - Method in class org.basmati.workspace.ImageTypeAction


actionPerformed(ActionEvent) - Method in class org.basmati.workspace.ImportImages


actionPerformed(ActionEvent) - Method in class org.basmati.workspace.OpenImageRice


actionPerformed(ActionEvent) - Method in class org.basmati.workspace.ScaleImage


actionPerformed(ActionEvent) - Method in class org.basmati.workspace.VeiwParticlesAnchors


addImage(Image) - Method in class org.basmati.workspace.ImageViewScene


associateLookup() - Method in class org.basmati.workspace.EditandViewGrainFileActionDataObject


attachEdgeSourceAnchor(String, String, String) - Method in class org.basmati.workspace.GraphUI


attachEdgeSourceAnchor(String, Particle, Particle) - Method in class org.basmati.workspace.ParticleScene


attachEdgeTargetAnchor(String, String, String) - Method in class org.basmati.workspace.GraphUI


attachEdgeTargetAnchor(String, Particle, Particle) - Method in class org.basmati.workspace.ParticleScene


attachEdgeWidget(String) - Method in class org.basmati.workspace.GraphUI


attachEdgeWidget(String) - Method in class org.basmati.workspace.ParticleScene


attachNodeWidget(String) - Method in class org.basmati.workspace.GraphUI


attachNodeWidget(Particle) - Method in class org.basmati.workspace.ParticleScene


## C

calcpb() - Method in class org.basmati.workspace.GraphUI
:   Calculate the main level branches

calcsb() - Method in class org.basmati.workspace.GraphUI
:   Calculate the second level branches

calctb() - Method in class org.basmati.workspace.GraphUI
:   Calculate the third level branches

checkResults() - Method in class org.basmati.workspace.ParticleScene


clear() - Method in class org.basmati.workspace.GraphUI


clear() - Method in class org.basmati.workspace.ParticleScene


CollectMasterReportAction - Class in org.basmati.workspace
:   The action handler when the user click on the collect report button.

CollectMasterReportAction(RiceProject) - Constructor for class org.basmati.workspace.CollectMasterReportAction


ColoredImageAction - Class in org.basmati.workspace
:   The handler of of the "colored image" button action.

ColoredImageAction() - Constructor for class org.basmati.workspace.ColoredImageAction


componentActivated() - Method in class org.basmati.workspace.ParticlesEditorTopComponent


componentClosed() - Method in class org.basmati.workspace.GraphEditorTopComponent


componentClosed() - Method in class org.basmati.workspace.ParticlesEditorTopComponent


componentClosed() - Method in class org.basmati.workspace.ResultsEditorTopComponent


componentOpened() - Method in class org.basmati.workspace.GraphEditorTopComponent


componentOpened() - Method in class org.basmati.workspace.ParticlesEditorTopComponent


componentOpened() - Method in class org.basmati.workspace.ResultsEditorTopComponent


countMainGeneratingPoints(ArrayList<CircleWidget>) - Method in class org.basmati.workspace.GraphUI


createEditor(Lookup) - Static method in class org.basmati.workspace.EditandViewGrainFileActionDataObject


createFile(GraphModel, String, String, String) - Static method in class org.basmati.workspace.ResultProducer
:   Given memory objects, this function writes a single result xml file that
    holds all information

createFile(GraphModel, String, File) - Static method in class org.basmati.workspace.ResultProducer
:   Given memory objects, this function writes a single result xml file that
    holds all information

createFile(ParticlesModel, String, String, String) - Static method in class org.basmati.workspace.ResultProducer
:   Write down the input particle model to an XML file.

createFile(ParticlesModel, String, File) - Static method in class org.basmati.workspace.ResultProducer
:   Write down the input particle model to an XML file.

createLabel() - Method in class org.basmati.workspace.ImageViewScene


createNodeDelegate() - Method in class org.basmati.workspace.ProcessedRiceImageFileDataObject


createSelectionSquare(Rectangle, int) - Method in class org.basmati.workspace.ImageViewScene
:   Create the selection frame


## D

deepCopy(BufferedImage) - Static method in class org.basmati.workspace.Drawer
:   Create a copy of a given buffered image

Drawer - Class in org.basmati.workspace
:   Superimpose graph and particles on a given image

Drawer() - Constructor for class org.basmati.workspace.Drawer


drawGraph(Image, GraphModel) - Static method in class org.basmati.workspace.Drawer
:   Super impose a graph on an image

DrawGraphModel() - Method in class org.basmati.workspace.GraphUI
:   This function takes a Graphmodel and convert it to an editable graph on the scene

drawParticlesModel(Image, ParticlesModel, Color, boolean) - Static method in class org.basmati.workspace.Drawer
:   Draw a set of particles on a given image, the blue color is used.


## E

EditandViewGrainFileActionDataObject - Class in org.basmati.workspace
:   The data object implementation to represent the grain result file.

EditandViewGrainFileActionDataObject(FileObject, MultiFileLoader) - Constructor for class org.basmati.workspace.EditandViewGrainFileActionDataObject


EditResultFileAction - Class in org.basmati.workspace
:   The handler of the view/edit result file action.

EditResultFileAction(DataObject) - Constructor for class org.basmati.workspace.EditResultFileAction


EditViewGrains - Class in org.basmati.workspace
:   The handle of the view/edit grain action.

EditViewGrains(DataObject) - Constructor for class org.basmati.workspace.EditViewGrains


ExpressRunAction - Class in org.basmati.workspace
:   The handler of the run express command in the tool bar.

ExpressRunAction(RiceProject) - Constructor for class org.basmati.workspace.ExpressRunAction


## F

findgrains(FileObject) - Static method in class org.basmati.workspace.GrainsFinder
:   Detect the grains in the branches.

findgrains(FileObject[]) - Static method in class org.basmati.workspace.GrainsFinder
:   Detect the grains of a set images.

FindGrainsAction - Class in org.basmati.workspace
:   The handler of finding all grains in all image in the selected project.

FindGrainsAction(RiceProject) - Constructor for class org.basmati.workspace.FindGrainsAction


FindGrainsSingleImageAction - Class in org.basmati.workspace
:   The handler of detecting the grains in a single image action from the context menu.

FindGrainsSingleImageAction(DataObject) - Constructor for class org.basmati.workspace.FindGrainsSingleImageAction


findGrainsTraits(FileObject) - Static method in class org.basmati.workspace.GrainTraitsFinder
:   Detect the grains in a single image file.

findGrainsTraits(FileObject[]) - Static method in class org.basmati.workspace.GrainTraitsFinder
:   Detect the grains in a set of images and store the results to XML files.

FindGrainTraitsAction - Class in org.basmati.workspace
:   The handler of the find grain traits action.

FindGrainTraitsAction(RiceProject) - Constructor for class org.basmati.workspace.FindGrainTraitsAction


FindGrainTraitsSingleImage - Class in org.basmati.workspace
:   The handler of the find grain trait action for a single image from the
    context menu.

FindGrainTraitsSingleImage(DataObject) - Constructor for class org.basmati.workspace.FindGrainTraitsSingleImage


FindSkeletonAction - Class in org.basmati.workspace
:   The handler of finding the structure of all images in the selected project.

FindSkeletonAction(RiceProject) - Constructor for class org.basmati.workspace.FindSkeletonAction


findStructure(FileObject) - Static method in class org.basmati.workspace.StructureFinder
:   Find the structure in a single file.

findStructure(FileObject[]) - Static method in class org.basmati.workspace.StructureFinder
:   Find the structure of different files.

FindStructureSingleImageAction - Class in org.basmati.workspace
:   The handler of finding the structure in the selected image

FindStructureSingleImageAction(DataObject) - Constructor for class org.basmati.workspace.FindStructureSingleImageAction


fit2view(JScrollPane) - Method in class org.basmati.workspace.GraphUI


fit2view(JScrollPane) - Method in class org.basmati.workspace.ParticleScene


## G

generatReports(FileObject[][], RiceProject) - Static method in class org.basmati.workspace.ReportGenerator
:   Generate all report for all result files (structure and grains).

getAmount() - Method in class org.basmati.workspace.ScaleSelectorPanel


getbestNeighbor(CircleWidget, GraphUI.calcType) - Method in class org.basmati.workspace.GraphUI
:   In graph decomposition this function searches for the "winner" vertex to be followed during the decomposition.

getGm() - Method in class org.basmati.workspace.GraphUI


getGravity() - Method in class org.basmati.workspace.GraphUI


getHelpCtx() - Method in class org.basmati.workspace.ColoredImageAction


getHelpCtx() - Method in class org.basmati.workspace.ImageTypeAction


getHelpCtx() - Method in class org.basmati.workspace.VeiwParticlesAnchors


getImagefile() - Method in class org.basmati.workspace.GraphUI


getImagefile() - Method in class org.basmati.workspace.ParticleScene


getleftmost(ArrayList<CircleWidget>) - Method in class org.basmati.workspace.GraphUI


getLookup() - Method in class org.basmati.workspace.ProcessedRiceImageFileDataObject


getMainProject() - Static method in class org.basmati.workspace.ProjectUIManager


getMeanBranchesLength() - Method in class org.basmati.workspace.GraphUI


getMenuPresenter() - Method in class org.basmati.workspace.ColoredImageAction


getMenuPresenter() - Method in class org.basmati.workspace.ImageTypeAction


getMenuPresenter() - Method in class org.basmati.workspace.VeiwParticlesAnchors


getModel() - Method in class org.basmati.workspace.ParticleScene


getName() - Method in class org.basmati.workspace.ColoredImageAction


getName() - Method in class org.basmati.workspace.ImageTypeAction


getName() - Method in class org.basmati.workspace.VeiwParticlesAnchors


getNeighbors(CircleWidget) - Method in class org.basmati.workspace.GraphUI
:   A helper function to get the neighbors of a given widget

getneighborsofColor(ArrayList<CircleWidget>, Color) - Method in class org.basmati.workspace.GraphUI


getneighborsofColor(ArrayList<CircleWidget>, Color, Color) - Method in class org.basmati.workspace.GraphUI


getneighborsofColor(ArrayList<CircleWidget>, Color, Color, Color, CircleWidget) - Method in class org.basmati.workspace.GraphUI


getneighborsofColor(ArrayList<CircleWidget>, Color, CircleWidget) - Method in class org.basmati.workspace.GraphUI


getOriginalImage() - Method in class org.basmati.workspace.ParticleScene


getrightmost(ArrayList<CircleWidget>) - Method in class org.basmati.workspace.GraphUI


getScaleRatio() - Method in class org.basmati.workspace.ScalingPanel
:   This method is called from within the constructor to initialize the form.

getSelectionBounds(int) - Method in class org.basmati.workspace.ImageViewScene


getStartandEndPoints() - Method in class org.basmati.workspace.GraphUI
:   Get start and end points

getToolbarPresenter() - Method in class org.basmati.workspace.ColoredImageAction


getToolbarPresenter() - Method in class org.basmati.workspace.ImageTypeAction


getToolbarPresenter() - Method in class org.basmati.workspace.VeiwParticlesAnchors


getUndoRedo() - Method in class org.basmati.workspace.ParticlesEditorTopComponent


getUpdatedParticles() - Method in class org.basmati.workspace.ParticleScene


getVertexGravity(CircleWidget, GraphUI.calcType) - Method in class org.basmati.workspace.GraphUI
:   Calculate the weight of the vertex.

GrainsFinder - Class in org.basmati.workspace
:   Grains finder finds the grains in both a single image and a set of images.

GrainsFinder() - Constructor for class org.basmati.workspace.GrainsFinder


GrainTraitsFinder - Class in org.basmati.workspace
:   The grain's traits finder class works for both a single image and a set of
    images.

GrainTraitsFinder() - Constructor for class org.basmati.workspace.GrainTraitsFinder


GraphEditorTopComponent - Class in org.basmati.workspace
:   Top component which displays the source images.

GraphEditorTopComponent() - Constructor for class org.basmati.workspace.GraphEditorTopComponent


GraphUI - Class in org.basmati.workspace
:   The GraphScene which shows the structure as a graph.

GraphUI() - Constructor for class org.basmati.workspace.GraphUI


GraphUI.calcType - Enum in org.basmati.workspace
:   This is the type of the calculation required on the graph


## H

highlightPaths(Vertex) - Method in class org.basmati.workspace.GraphUI
:   Deprecated.


## I

iconResource() - Method in class org.basmati.workspace.ColoredImageAction


iconResource() - Method in class org.basmati.workspace.ImageTypeAction


iconResource() - Method in class org.basmati.workspace.VeiwParticlesAnchors


ImageScaler - Class in org.basmati.workspace
:   The scaler of the input images in the selected project.

ImageScaler() - Constructor for class org.basmati.workspace.ImageScaler


ImageTypeAction - Class in org.basmati.workspace
:   The handler of the image background type option.

ImageTypeAction() - Constructor for class org.basmati.workspace.ImageTypeAction


ImageViewScene - Class in org.basmati.workspace
:   The Scene that shows the input images.

ImageViewScene() - Constructor for class org.basmati.workspace.ImageViewScene


ImportImages - Class in org.basmati.workspace
:   Handel the import image action.

ImportImages(Project) - Constructor for class org.basmati.workspace.ImportImages


increamental() - Method in class org.basmati.workspace.GraphUI


isallwithColorX(ArrayList<CircleWidget>, Color) - Method in class org.basmati.workspace.GraphUI


isDirty() - Method in class org.basmati.workspace.GraphUI


isDirty() - Method in class org.basmati.workspace.ParticleScene


isMainJunction(CircleWidget, ArrayList<CircleWidget>) - Method in class org.basmati.workspace.GraphUI


## M

mapColor2Type(Color) - Method in class org.basmati.workspace.GraphUI
:   Maps the input color to a vertex type.

mapStructureGrainsFiles(FileObject[]) - Method in class org.basmati.workspace.CollectMasterReportAction
:   Given a set of grain and structure result files, this function maps them to a (n/2)\*2 matrix of structure (in the first column) and grain (in the second column).

mapType2Color(Vertex.VERTEX\_TYPE) - Method in class org.basmati.workspace.GraphUI
:   Maps the input vertex type to a color

markTransitions() - Method in class org.basmati.workspace.GraphUI
:   Detect if there are any transition vertices in the graph.

measureLength() - Method in class org.basmati.workspace.GraphUI
:   Deprecated.

    *Now the user define the scale in the source image at the `ImageViewScene` class.*


## N

neighborsOfNumberX(ArrayList<CircleWidget>, Color) - Method in class org.basmati.workspace.GraphUI


neighborsOfNumberX(ArrayList<CircleWidget>, Color, Color) - Method in class org.basmati.workspace.GraphUI


## O

openImage(FileObject) - Method in class org.basmati.workspace.GraphEditorTopComponent


OpenImageRice - Class in org.basmati.workspace
:   The Handler when the user opens a single image in the image viewer.

OpenImageRice(DataObject) - Constructor for class org.basmati.workspace.OpenImageRice


org.basmati.workspace - package org.basmati.workspace


## P

ParticleScene - Class in org.basmati.workspace
:   The `GraphScene` which shows the grains and allows the user to edit
    them.

ParticleScene() - Constructor for class org.basmati.workspace.ParticleScene


ParticlesEditorTopComponent - Class in org.basmati.workspace
:   Top component which displays the grains and allows editing them.

ParticlesEditorTopComponent() - Constructor for class org.basmati.workspace.ParticlesEditorTopComponent


ProcessedRiceImageFileDataObject - Class in org.basmati.workspace
:   A `MultiDataObject` class that represents the processed image.

ProcessedRiceImageFileDataObject(FileObject, MultiFileLoader) - Constructor for class org.basmati.workspace.ProcessedRiceImageFileDataObject


ProjectUIManager - Class in org.basmati.workspace
:   The project handler class.

ProjectUIManager() - Constructor for class org.basmati.workspace.ProjectUIManager


## R

ReportGenerator - Class in org.basmati.workspace
:   The Reports generator.

ReportGenerator() - Constructor for class org.basmati.workspace.ReportGenerator


reset() - Method in class org.basmati.workspace.GraphUI


restallLabels() - Method in class org.basmati.workspace.GraphUI


ResultProducer - Class in org.basmati.workspace
:   It saver of all result files.

ResultProducer() - Constructor for class org.basmati.workspace.ResultProducer


ResultsEditorTopComponent - Class in org.basmati.workspace
:   Top component which displays the results of the structure detection.

ResultsEditorTopComponent() - Constructor for class org.basmati.workspace.ResultsEditorTopComponent


## S

scale(FileObject[], double) - Static method in class org.basmati.workspace.ImageScaler
:   Scale a set of images by a given scale factor.

ScaleImage - Class in org.basmati.workspace
:   The handler of the images scaling action.

ScaleImage(RiceProject) - Constructor for class org.basmati.workspace.ScaleImage


ScaleSelectorPanel - Class in org.basmati.workspace
:   The dialog of scaling the image.

ScaleSelectorPanel() - Constructor for class org.basmati.workspace.ScaleSelectorPanel
:   Creates new form ScaleSelectorPanel

ScalingPanel - Class in org.basmati.workspace
:   The panel of the scaling dialog

ScalingPanel() - Constructor for class org.basmati.workspace.ScalingPanel
:   Creates new form ScalingPanel

scene - Variable in class org.basmati.workspace.ParticlesEditorTopComponent


selectScale(long) - Method in class org.basmati.workspace.ImageViewScene
:   Set the scale on the input image.

setcolor(ArrayList<CircleWidget>, Color) - Method in class org.basmati.workspace.GraphUI


setDirty(boolean) - Method in class org.basmati.workspace.GraphUI


setDirty(boolean) - Method in class org.basmati.workspace.ParticleScene


setGm(GraphModel) - Method in class org.basmati.workspace.GraphUI


setImagefile(FileObject) - Method in class org.basmati.workspace.GraphUI


setImagefile(File) - Method in class org.basmati.workspace.ParticleScene


setModel(ParticlesModel) - Method in class org.basmati.workspace.ParticleScene


setOriginalImage(Image) - Method in class org.basmati.workspace.ParticleScene


setPixels(String) - Method in class org.basmati.workspace.ScaleSelectorPanel


setupActions() - Method in class org.basmati.workspace.ImageViewScene


setupImageWidget() - Method in class org.basmati.workspace.ParticleScene


setupLayers(Image) - Method in class org.basmati.workspace.GraphUI


setuplayers() - Method in class org.basmati.workspace.ImageViewScene


setuplayers() - Method in class org.basmati.workspace.ParticleScene


setupLogicalModels(GraphModel, FileObject) - Method in class org.basmati.workspace.GraphUI


setUpmodel() - Method in class org.basmati.workspace.ParticleScene


StructureFinder - Class in org.basmati.workspace
:   This class detects the structure of the panicle either in a single or
    multiple images.

StructureFinder() - Constructor for class org.basmati.workspace.StructureFinder


swapXY(Particle) - Method in class org.basmati.workspace.ParticleScene


## U

updateGraphModel() - Method in class org.basmati.workspace.GraphUI
:   Update the graph model when user have made some changes.

updateImagewidget() - Method in class org.basmati.workspace.ParticleScene


updateParticlesModel(boolean) - Method in class org.basmati.workspace.ParticleScene
:   Update the particles model in order to save it to a file

UpdateScene() - Method in class org.basmati.workspace.ParticlesEditorTopComponent


## V

valueOf(String) - Static method in enum org.basmati.workspace.GraphUI.calcType
:   Returns the enum constant of this type with the specified name.

values() - Static method in enum org.basmati.workspace.GraphUI.calcType
:   Returns an array containing the constants of this enum type, in
    the order they are declared.

VeiwParticlesAnchors - Class in org.basmati.workspace
:   The handler of toggling the anchor points of the grains ON and OFF.

VeiwParticlesAnchors() - Constructor for class org.basmati.workspace.VeiwParticlesAnchors

A C D E F G H I M N O P R S U V

- Package
- Class
- Tree
- Deprecated
- Index
- Help

*org.basmati.workspace 1.0*

- Prev
- Next

- Frames
- No Frames

- All Classes
